# Fine Mapping of Two Major Quantitative Trait Loci for Rice Chalkiness With High Temperature-Enhanced Additive Effects

**DOI:** 10.3389/fpls.2022.957863

**Published:** 2022-06-30

**Authors:** Weifeng Yang, Qingwen Hao, Jiayan Liang, Quanya Tan, Xin Luan, Shaojun Lin, Haitao Zhu, Suhong Bu, Zupei Liu, Guifu Liu, Shaokui Wang, Guiquan Zhang

**Affiliations:** ^1^Guangdong Provincial Key Laboratory of Plant Molecular Breeding, State Key Laboratory for Conservation and Utilization of Subtropical Agro-Bioresources, South China Agricultural University, Guangzhou, China; ^2^Guangdong Laboratory for Lingnan Modern Agriculture, Guangzhou, China

**Keywords:** chalkiness, QTL, SSSL, substitution mapping, high temperature, rice quality

## Abstract

Chalkiness is a crucial determinant of rice quality. During seed filling period, high temperature usually increases grain chalkiness, resulting in poor grain quality. Rice chalkiness was controlled by quantitative trait loci (QTLs) and influenced by environmental conditions. In this study, we identified two single-segment substitution lines (SSSLs) 22–05 and 15–06 with significantly lower percentage of grain chalkiness (PGC) than recipient Huajingxian 74 (HJX74) over 6 cropping seasons. Two major QTLs for chalkiness, *qPGC5* and *qPGC6*, were located by substitution mapping of SSSLs 22–05 and 15–06, respectively. *qPGC5* was located in the 876.5 kb interval of chromosome 5 and *qPGC6* was located in the 269.1 kb interval of chromosome 6. Interestingly, the PGC of HJX74 was significantly different between the two cropping seasons per year, with 25.8% in the first cropping season (FCS) and 16.6% in the second cropping season (SCS), while the PGC of SSSLs 22–05 and 15–06 did not significantly differ between FCS and SCS. The additive effects of *qPGC5* and *qPGC6* on chalkiness in the SSSLs were significantly greater in FCS than in SCS. These results showed that *qPGC5* and *qPGC6* had major effects on chalkiness and the SSSL alleles were more effective in reducing chalkiness under high temperature condition in FCS. The fine-mapping of the two QTLs will facilitate the cloning of genes for chalkiness and provide new genetic resources to develop new cultivars with low chalkiness even under high temperature condition.

## Introduction

Rice (*Oryza sativa*) is an essential cereal crop in the world. Rice cultivars of high yield and good quality are required to meet human needs. Grain appearance is a crucial trait to determine rice quality. Chalk is an opaque area of the seed endosperm, which affects grain appearance and milling and cooking performance (Fitzgerald et al., 2009; Misra et al., 2019). Rice chalkiness is easily affected by environments. During seed development, high temperature stress gives rise to irregular grain-filling and obstacles to storage biosynthesis, resulting in the emergence of chalkiness (Sreenivasulu et al., 2015; Edwards et al., 2017; Nevame et al., 2018). The ratio of source to sink had a significant effect on chalkiness, and the degree of influence varied with cultivars (Cheng et al., 2007). By comparison, the chalkiness level of new cultivars is higher than that of modern old cultivars, while hybrid cultivars are usually higher than other modern cultivars (Laborte et al., 2015). Consequently, high-yield cultivars usually exhibit high chalkiness (Misra et al., 2019).

Rice chalkiness is a complex trait controlled by multiple quantitative trait loci (QTLs) and is quantified as the percentage of grain chalkiness (PGC) (Sreenivasulu et al., 2015; Misra et al., 2019; Yang et al., 2021a). In the past decades, a large number of QTLs for rice chalkiness have been detected using diverse mapping populations (Sreenivasulu et al., 2015; Yang et al., 2021a). It was found that rice chromosomes 5 and 6 were hotspot regions for chalkiness QTLs (Tan et al., 2000; Wan et al., 2005; Zhou et al., 2009; Chen et al., 2011, 2016; Liu et al., 2012; Peng et al., 2014; Gao et al., 2016; Yun et al., 2016; Zhao et al., 2016; Wang et al., 2017; Misra et al., 2020). Some QTLs for high temperature-induced chalkiness were detected using heat stress-sensitive cultivars, and these QTLs accelerated the occurrence of chalkiness under high temperature conditions (Yamakawa et al., 2008; Kobayashi et al., 2013; Wada et al., 2015; Nevame et al., 2018; Yang et al., 2021a). Until now, a few QTLs were mapped in a fine resolution or cloned (Wu et al., 2021; Yang et al., 2021a). Li et al. (2014) cloned a major QTL positively regulating rice chalkiness, *Chalk5*, which encodes a vacuolar H^–^-translocating pyrophosphatase (V-PPase) and is specially expressed in endosperm. Most rice cultivars have higher *Chalk5* expression levels and higher grain chalkiness. Yang et al. (2022) identified a transcription factor *OsbZIP60* controlling grain chalkiness by using genome-wide association studies (GWAS). The moderate expression of *OsbZIP60* can maintain endoplasmic reticulum (ER) homeostasis. The impaired function of *OsbZIP60* disturbs the ER homeostasis, and then *OsbZIP50*-mediated unfolded protein response activation is triggered dealt with to ER stress, thus affecting the expression of genes for endosperm development, finally resulting in chalkiness formation. Wu et al. (2022) cloned the *WCR1* on chromosome 1, which negatively regulates rice chalkiness. Two alleles *WCR1^A^* and *WCR1^G^* were identified based on the SNP-A/G in the promoter region of *WCR1*. OsDOF17, as a transcriptional activator, promotes the transcription of *WCR1^A^*. And then, WCR1 interacts with metallothionein MT2b to maintain redox homeostasis in the endosperm of rice, thereby reducing grain chalkiness.

Chalkiness occurs in the endosperm of seeds and is influenced by some seed development genes. The dysfunctional *GW2* allele was shown to enhance width and weight of rice grain, while causing the increase in grain chalkiness (Song et al., 2007). The alleles *GW7*, *gw8* and *gs9* caused much slenderer grain shape, resulting in the reducing of rice chalkiness (Wang et al., 2012, 2015; Zhao et al., 2018). Recently, Jiang et al. (2021) identified *GSE5* had a pleiotropic function to regulating grain shape and chalkiness by map-based cloning. Several genes for endosperm development have been found to influence grain-filling and chalkiness. *OsPPDKB* regulated carbon metabolism during grain filling, which mutant showed white-core endosperm (Kang et al., 2005). *SSIIIa* had pleiotropic effects on endosperm development, which mutant had a chalky interior appearance in seeds (Fujita et al., 2007). *GIF1* encoded a cell-wall invertase required for carbon partitioning in the early stage of grain-filling, which mutant exhibited abnormal development and loose accumulation of starch granules, resulting in the increase of grain chalkiness (Wang et al., 2008). *UGPase1* had an effect on male sterility and grain chalkiness (Woo et al., 2008). Some *floury endosperm* (*flo*) mutants including *flo2*, *flo7*, *flo10* and *flo19* caused the obstacles to storage biosynthesis and amyloplast development, leading to the formation of floury endosperm (She et al., 2010; Zhang et al., 2016; Wu et al., 2019; Lou et al., 2021). Interestingly, many chalkiness QTLs are located in the same region as other seed development genes (Zhao et al., 2015; Yang et al., 2021b). These results indicate that rice chalkiness is a complex trait, and is affected by the pleiotropic effect of other genes.

Recently, we identified four QTLs for rice chalkiness using the single-segment substitution lines (SSSLs) with the Huajingxian 74 (HJX74) genetic background (Yang et al., 2021a,b). Two QTLs, *qPGC9* on chromosome 9 and *qPGC11* on chromosome 11, were sensitive to high temperature, which reduced their additive effect on chalkiness in the cropping season of high temperature (Yang et al., 2021a). By comparison, *qPGC8.1* and *qPGC8.2*, as two closely linked QTLs on chromosome 8, were insensitive to high temperature (Yang et al., 2021b). In this study, two QTLs for rice chalkiness, *qPGC5* and *qPGC6*, were detected by substitution mapping using HJX74-SSSLs. Compared with the four QTLs detected previously, *qPGC5* and *qPGC6* had major effects on grain chalkiness, and their SSSL alleles had the additive effects enhanced by high temperature. The fine-mapping of *qPGC5* and *qPGC6* will contribute to understand the genetic architecture of rice chalkiness and to develop the cultivars with low chalkiness even under high temperature.

## Materials and Methods

### Rice Materials and Field Cultivation

Two SSSLs 22–05 and 15–06 with low rice chalkiness were selected from the HJX74-SSSL library (Zhang, 2019, 2021). The substitution segment of 22–05 was from Khazar, a *japonica* variety, and that of 15–06 was from American Jasmine, an *indica* variety. Rice materials were grown in the experimental field of South China Agricultural University (23°07′N, 113°15′E), Guangzhou in 2017–2019 with two cropping seasons per year. The first cropping season (FCS) was from late February to mid-July and the second cropping season (SCS) from late July to mid-November. Rice planting and pest control followed conventional practices (Yang et al., 2021a).

### Phenotyping of Traits

The seeds of 10 plants per line were harvested after full maturity. The dry seeds per plant were separately processed into milled rice, and 200 head milled rice per plant were used to measure chalkiness (Yang et al., 2021a). The percentage of chalky grains (PCG) per plant and the percentage of chalky area (PCA) per chalky grain were measured by a rice quality analyzer.^1^ PGC is the product of PCG times PCA (Yang et al., 2021a). The investigations of heading date, panicle number per plant and plant height were carried out in the experimental field. The measurements of grain yield traits were performed using the yield traits scorer, a rice phenotyping facility (Yang et al., 2014).

### Genotyping and Substitution Mapping

“RM” markers were selected from Gramene database.^2^ New markers used in this study were designed using the sequences of insertions/deletions (InDels) or SNPs between recipient HJX74 and donors by the software of Primer Premier 5.0 (Supplementary Table 1). PCR products were separated on 6% denaturing PAGE and detected by silver staining (Tan et al., 2020). The secondary SSSLs or near-isogenic lines (NILs) were developed from the crosses between SSSL 22–05 or 15–06 and recipient HJX74. The lengths of substitution segments were estimated by the marker position on the substitution segments (Tan et al., 2020). When the PGC of SSSL or NIL was significantly different from that of HJX74, a QTL for PGC was located on the substitution segment of SSSL or NIL. When multiple NILs with overlapping substitution segments showed significantly different phenotypes from HJX74, the QTL was detected in the overlapping region (Eshed and Zamir, 1995; Tan et al., 2020).

Linkage maps of markers were drawn by MapChart2.3. Additive effect of a QTL was estimated by the phenotype difference between NIL and HJX74 (Zhou et al., 2020). QTL naming followed the proposed rules (McCouch et al., 1997).

### Statistical Analysis

The comparison of the two sets of data was performed by Student’s *t*-test. The comparisons between multiple groups and the control group were performed by Dunnett *t*-test. The multiple range test among multiple groups was done by least significance range (LSR) (Duncan, 1955). For the statistical analysis, data of percentages were converted to the arcsine square root. SPSS statistics 23.0 and OriginPro 9.0 were used for the data analysis and figure making.^3^

## Results

### Rice Chalkiness in Single-Segment Substitution Lines

**FIGURE 1:**
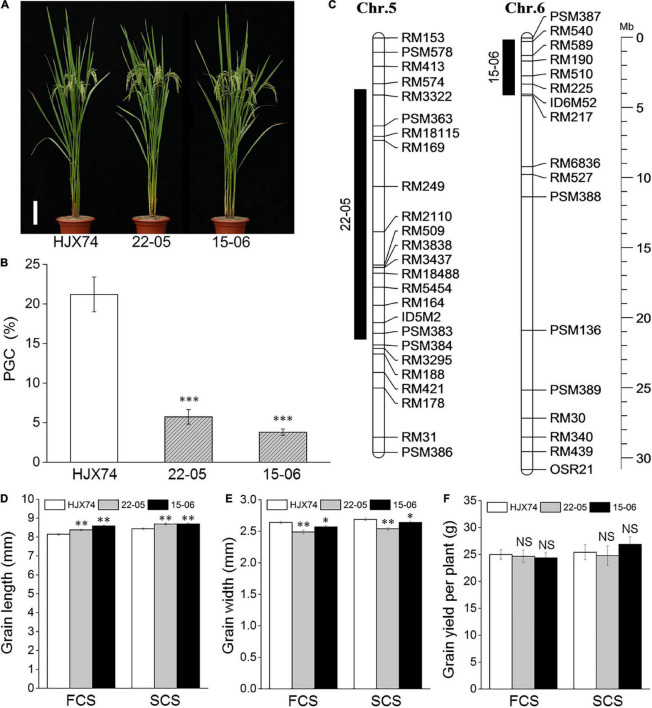
Phenotypes and substitution segments of two SSSLs. **(A)** Plant type of SSSLs 22–05 and 15–06 and control HJX74. Scale bar, 15 cm. **(B)** Percentage of grain chalkiness (PGC) of SSSLs and control HJX74. PGC is shown as mean ± S.E. in six cropping seasons. **(C)** Substitution segments of SSSLs 22–05 and 15–06. Black bars on the left of chromosomes represent the substitution segments of SSSLs. Physical distance is shown on the ruler. Chr., Chromosome. Mb, Megabase. The grain length **(D)**, grain width **(E)** and grain yield per plant **(F)** of SSSLs 22–05 and 15–06 and control HJX74 are shown as mean ± S.E. FCS, First cropping season; SCS, Second cropping season. ^∗^, ^∗∗^ and ^∗∗∗^, Significant difference at the 0.05, 0.01, and 0.001 levels, respectively. NS, No significance.

Two SSSLs 22–05 and 15–06 were used to measure rice chalkiness over the 6 cropping seasons from 2017 to 2019 (Figure 1A). Compared with recipient HJX74, the PCG, PCA and PGC of 22–05 and 15–06 were significantly reduced in every cropping season (Supplementary Table 2). On average, the PGC of 22–05 and 15–06 were 5.7 and 3.8%, respectively, much lower than 21.2% of recipient HJX74 (Figure 1B and Supplementary Table 2). The results showed that there were QTLs for rice chalkiness in the substitution segments of the two SSSLs. The substitution segments of 22–05 and 15–06 were detected by markers. The estimated lengths of substitution segments were 17.81 Mb in 22–05 and 3.92 Mb in 15–06 (Figure 1C and Supplementary Table 3).

Eight agronomic traits of 22–05 and 15–06 were surveyed in two cropping seasons. Compared with HJX74, panicle number per plant, total grain number per plant and grain yield per plant of 22–05 and 15–06, and plant height and 1,000-grain weight of 15–06 had no significant difference, while heading date, grain length and width of 22–05 and 15–06, and plant height and 1,000-grain weight of 22–05 showed significant difference in two cropping seasons or in only one cropping season (Figures 1A,D–F and Supplementary Table 4). The result indicated that the SSSLs and HJX74 had a similar genetic background.

### Substitution Mapping of *qPGC5*

**FIGURE 2:**
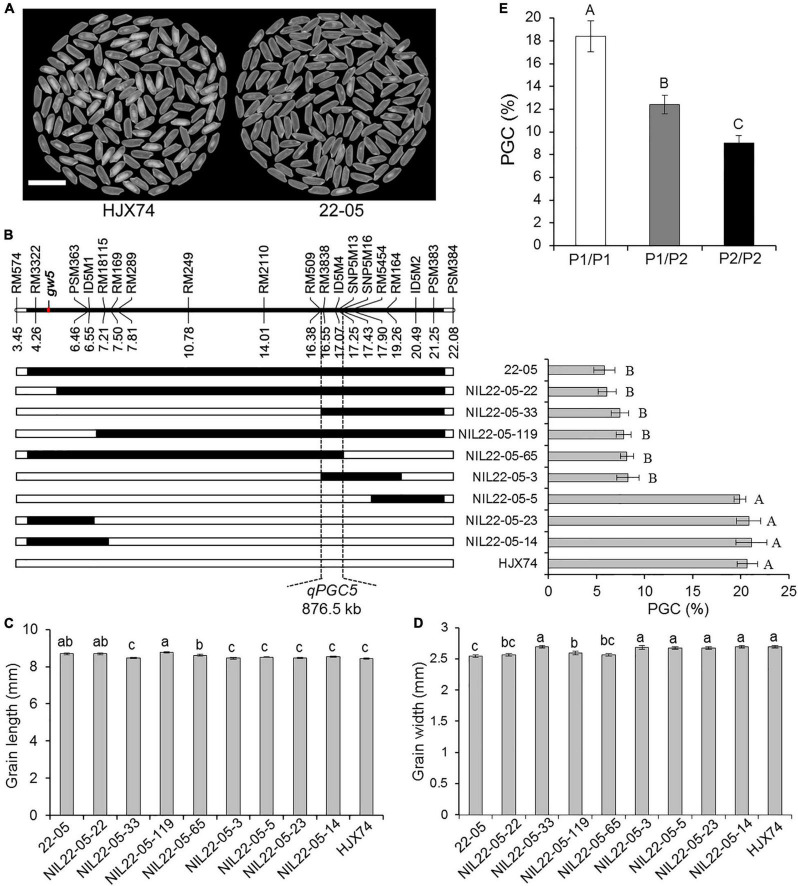
Substitution mapping of *qPGC5.* **(A)** The appearance of milled rice of SSSL 22–05 and control HJX74. Scale bar, 1 cm. **(B)** Substitution mapping of *qPGC5* in SSSL 22–05. The positions of markers on the chromosome are shown in physical distance (Mb). The position of *gw5* gene is shown on the chromosome. Black blocks show the substitution segments from 22–05 in NILs. PGC (%) is the mean ± S.E. of two cropping seasons. Grain length **(C)** and width **(D)** of 22–05 and NILs are given as the mean ± S.E. of two cropping seasons. **(E)** PGC of three *qPGC5* genotypes in an F_2_ population. P1/P1, The genotype of *qPGC5-HJX74/qPGC5-HJX74* (*n* = 25); P1/P2, The genotype of *qPGC5-HJX74/qPGC5-NIL* (*n* = 48); P2/P2, The genotype of *qPGC5-NIL/qPGC5-NIL* (*n* = 27). Different uppercase letters and lowercase letters indicate significant differences at the 0.05 level and 0.01 level, respectively.

In order to map the QTL controlling rice chalkiness in the substitution segment of SSSL 22–05, the SSSL was used to produce secondary SSSLs or NILs. Eight NILs were obtained from an F_2:3_ population of the HJX74/22-05 cross. Then the rice chalkiness of the eight NILs was measured in two cropping seasons. PGC levels of five NILs, NIL22-05-22, NIL22-05-33, NIL22-05-119, NIL22-05-65, and NIL22-05-3, were as low as 22–05, while those of other three NILs, NIL22-05-5, NIL22-05-23, and NIL22-05-14, were as high as HJX74. In the region from RM509 to SNP5M16, the substitution segments of the five NILs with low PGC overlapped each other, while the substitution segments of the other three NILs with high PGC were outside this region. The result showed that the QTL controlling the PGC, *qPGC5*, was mapped in the estimated region of 876.5 kb between markers RM509 and SNP5M16 (Figures 2A,B). In addition, the grain length and width of the NILs were segregated, but not co-segregated with the grain chalkiness controlled by *qPGC5* (Figures 2C,D). The grain width gene *gw5* was located on the substitution segment of 22–05, but far away from the *qPGC5* interval (Figure 2B).

Using marker ID5M4 in *qPGC5* interval, the segregation of marker genotypes in an F_2_ population of 100 plants was tested by Chi-square test. The results showed that the plants number of three marker genotypes were 25, 48, and 27, respectively, with a segregation ratio of 1:2:1 (χ^2^ = 2.27 < χ^2^_0.01, 2_ = 9.21). The PGC of heterozygous genotype *qPGC5-NIL*/*qPGC5-HJX74* was significantly higher than that of *qPGC5-NIL*/*qPGC5-NIL* genotype and significantly lower than that of *qPGC5-HJX74*/*qPGC5-HJX74* genotype. Therefore, *qPGC5* showed an incomplete dominance effect on grain chalkiness (Figure 2E).

### Substitution Mapping of *qPGC6*

**FIGURE 3:**
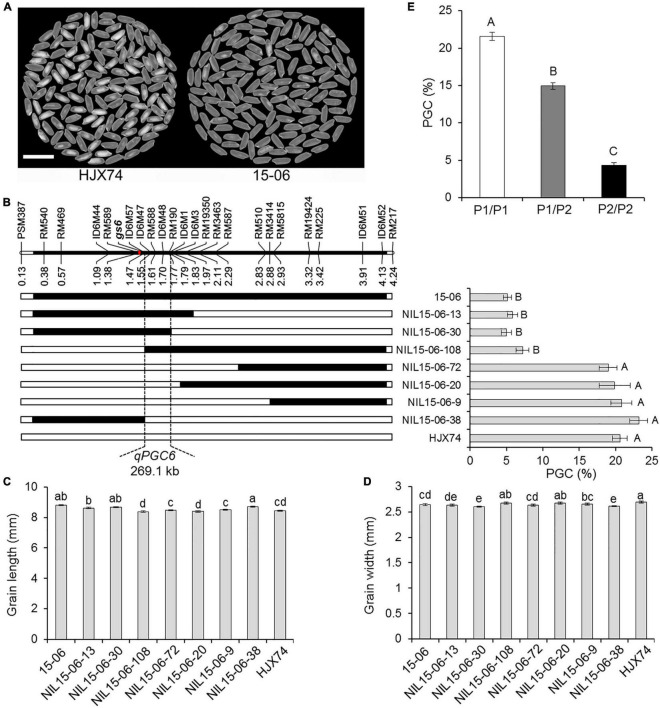
Substitution mapping of *qPGC6.* **(A)** The appearance of milled rice of SSSL 15–06 and control HJX74. Scale bar, 1 cm. **(B)** Substitution mapping of *qPGC6* in SSSL 15–06. The positions of markers on the chromosome are shown in physical distance (Mb). The position of *gs6* gene is shown on the chromosome. Black blocks show the substitution segments from 15–06 in NILs. PGC (%) is the mean ± S.E. of two cropping seasons. Grain length **(C)** and width **(D)** of 15–06 and NILs are given as the mean ± S.E. of two cropping seasons. **(E)** PGC of three *qPGC6* genotypes in an F_2_ population. P1/P1, The genotype of *qPGC6-HJX74/qPGC6-HJX74* (*n* = 45); P1/P2, The genotype of *qPGC6-HJX74/qPGC6-NIL* (*n* = 84); P2/P2, The genotype of *qPGC6-NIL/qPGC6-NIL* (*n* = 41). Different uppercase letters and lowercase letters indicate significant differences at the 0.05 level and 0.01 level, respectively.

In order to map the QTL controlling rice chalkiness on the substitution segment of SSSL 15–06, the SSSL was used to produce secondary SSSLs or NILs. Seven NILs were obtained from an F_2:3_ population of HJX74/15-06 cross. The rice chalkiness of the seven NILs was then measured in two cropping seasons. Three NILs, NIL15-06-13, NIL15-06-30, and NIL15-06-108, showed PGC levels as low as 15–06, while the other four NILs, NIL15-06-72, NIL15-06-20, NIL15-06-9, and NIL15-06-38 showed PGC levels as high as HJX74. In the region between markers ID6M57 and ID6M1, the substitution segments of the three NILs with low PGC overlapped each other, while the substitution segments of the other four NILs with high PGC were outside the region. The result showed that the chalkiness QTL, *qPGC6*, was mapped in the estimated interval of 269.1 kb between markers ID6M57 and ID6M1 (Figures 3A,B). In addition, the grain length and width of the NILs were segregated, but not co-segregated with the chalkiness controlled by *qPGC6* (Figures 3C,D). The grain size gene *gs6* was located on the substitution segment of 15–06, but outside the *qPGC6* interval (Figure 3B).

Using marker RM588 in *qPGC6* region, Chi-square test was carried out in an F_2_ population of 170 plants. The result showed that the plants number of three marker genotypes were 45, 84, and 41, respectively, which segregated in the ratio of 1:2:1 (χ^2^ = 1.40 < χ^2^_0.01, 2_ = 9.21). The PGC of heterozygous genotype *qPGC6-NIL*/*qPGC6-HJX74* differed markedly from those of the homozygous genotypes *qPGC6-NIL*/*qPGC6-NIL* and *qPGC6-HJX74*/*qPGC6-HJX74*. Therefore, *qPGC6* showed an incomplete dominance effect on grain chalkiness (Figure 3E).

### Influence of Cropping Seasons in Rice Chalkiness

**FIGURE 4:**
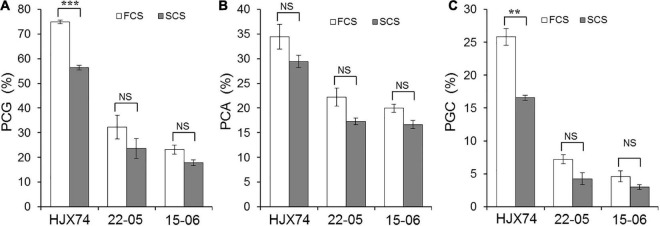
The chalkiness phenotypes of HJX74, 22–05 and 15–06 in the first cropping season (FCS) and the second cropping season (SCS). **(A)** PCG, Percentage of chalky grain. **(B)** PCA, Percentage of chalky area. **(C)** PGC, Percentage of grain chalkiness. ** and ***, Significant difference at the 0.01 and 0.001 levels, respectively. NS, No significance.

The rice materials were planted in two cropping seasons for chalkiness measurement per year. The daytime and nighttime temperatures during the period from flowering to harvest of rice were very different between FCS and SCS. In the FCS and SCS of 2017–2019, the maximum temperatures were 32.1 and 28.6°C, the minimum temperatures were 25.9 and 21.0°C, and the mean temperatures were 29.0 and 24.9°C, respectively. The average temperature in FCS increased by 4.1°C compared with that in SCS (Supplementary Table 5). In rice chalkiness, PGC of HJX74 was significantly different in two cropping seasons, which was 25.8% in FCS and 16.6% in SCS. In contrast, the PGC of SSSLs 22–05 and 15–06 did not differ significantly between FCS and SCS (Figure 4). The results indicated that, unlike HJX74, the chalkiness of 22–05 and 15–06 were not influenced by the high temperature of FCS.

PGC consists of PCG and PCA. The significant positive correlation of PCG and PCA with PGC was detected by regression correlation analysis. The regression coefficients of PCG, PCA, and PGC were 0.8232 and 0.6177 in 22–05 carrying *qPGC5*, and 0.9095 and 0.7112 in 15–06 carrying *qPGC6*, respectively (Supplementary Figure 1). The results showed that both PCG and PCA contributed greatly to PGC, and the contribution of PCG to PGC was greater than that of PCA.

### Additive Effects of *qPGC5* and *qPGC6* on Rice Chalkiness

**FIGURE 5:**
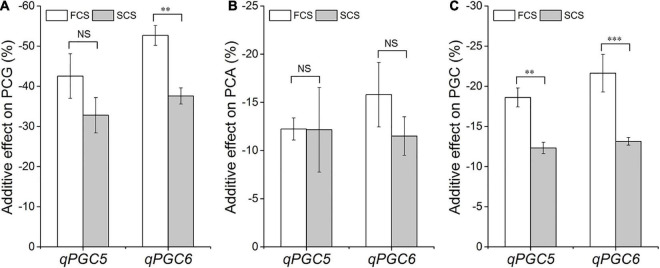
The additive effects of *qPGC5* and *qPGC6* on grain chalkiness in the first cropping season (FCS) and the second cropping season (SCS). **(A)** PCG, Percentage of chalky grain. **(B)** PCA, Percentage of chalky area. **(C)** PGC, Percentage of grain chalkiness. ^∗∗^ and ^∗∗∗^, Significant difference at the 0.01 and 0.001 levels, respectively. NS, No significance.

The additive effects of *qPGC5* and *qPGC6* were estimated from the PGC values of SSSLs and HJX74 in 2017–2019. The additive effects of *qPGC5* and *qPGC6* on chalkiness were negative and significantly different between FCS and SCS. The additive effect of *qPGC5* on PGC was –18.6% in FCS and –12.3% in SCS, respectively, and the additive effect in FCS was –6.3% greater than that in SCS. The additive effects of *qPGC6* on PGC in the FCS and SCS were –21.6 and –13.1%, respectively, and the additive effect in FCS increased by –8.5% compared with that in SCS (Figure 5). These results showed that *qPGC5* and *qPGC6* had major additive effects on chalkiness, and their additive effects in FCS were significantly greater than those in SCS. The high temperature of FCS enhanced the additive effects of *qPGC5* and *qPGC6*, making the QTLs more effective in decreasing rice chalkiness.

## Discussion

### *qPGC5* and *qPGC6* Were Mapped in the Hot-Spot Regions of Grain Development Genes

Many chalk QTLs were identified in both sides of centromere of rice chromosome 5 (Tan et al., 2000; Chen et al., 2011, 2016; Liu et al., 2012; Li et al., 2014; Gao et al., 2016; Yun et al., 2016; Zhao et al., 2016; Wang et al., 2017; Misra et al., 2020). Zhao et al. (2016) detected 32 (21) and 46 (22) QTLs for chalkiness based on single environment analysis in two sets of recombinant inbred lines (RILs) respectively, of which QTLs *qDEC5b* and *qPGWC5b* were located in the region of 14.22–18.35 Mb of chromosome 5. In this study, we located *qPGC5* in the region of 16.38–17.43 Mb (Figure 2), which overlaps with *qDEC5b* and *qPGWC5b*. On the short arm of chromosome 5, several genes related to grain development were identified. The major chalky QTL *Chalk5* was located in the position of 3.3 Mb of chromosome 5 (Li et al., 2014). Near *Chalk5*, *GS5* was responsible for grain size by adjusting grain width, grain filling and grain weight (Li et al., 2011). Located in about 5.4 Mb of the chromosome, *GW5* was a major QTL underlying grain width and grain weight, and regulated cell division through the brassinosteroid signaling pathway during seed development (Liu et al., 2017). Located ∼3 kb upstream to *GW5*, *chalk5.1*, a newly predicted gene identified by genome-wide association studies (GWAS), was responsible for chalkiness (Misra et al., 2019). These results showed that the regions flanking centromere of chromosome 5 are the hot-spots of chalkiness related QTLs.

Some QTLs on rice chalkiness were identified on short arm of chromosomes 6 in different experiments (Tan et al., 2000; Zhou et al., 2009; Chen et al., 2016; Gao et al., 2016; Yun et al., 2016; Zhao et al., 2016; Misra et al., 2020). Around the *Waxy* (*Wx*) gene controlling amylose content of endosperm (Wang et al., 1995), several chalkiness QTLs were identified. Tan et al. (2000) identified a QTL controlling the chalk of white core in the region of Wx-R1952. A putative QTL *qPGWC-6* for PGWC was detected close to the marker RM190, which is tightly linked to *Wx* gene (Temnykh et al., 2000; McCouch et al., 2002), in a chromosome segment substitution line (CSSL) population (Zhou et al., 2009). Misra et al. (2020) detected 78 QTL regions associated with chalkiness by GWAS, of which *PGC6.1* was located in the region of 1.22–1.87 Mb and *PGC6.2* in that of 2.03–2.23 Mb on the chromosome. In the present study, *qPGC6* was mapped in the region of 1.47–1.79 Mb, which includes the *Wx* locus (Figure 3). The results revealed that the region close to *Wx* locus on chromosome 6 is the hot spot of grain chalkiness QTLs.

### *qPGC5* and *qPGC6* Were Major Quantitative Trait Loci for Rice Chalkiness

Rice chalkiness is a quantitative trait. More than 100 QTLs have been identified, most of which have low genetic effect on rice chalkiness (Sreenivasulu et al., 2015; Wang et al., 2017; [Bibr B26][Bibr B22]; Yang et al., 2021a). Although 11 GWAS loci were identified to be responsible for chalky grain rate, they showed low heritability and minor effects for chalkiness (Gong et al., 2017). Using HJX74-SSSLs, we totally detected 6 QTLs for rice chalkiness in the same genetic background. The four QTLs detected previously, *qPGC9*, *qPGC11*, *qPGC8.1*, and *qPGC8.2*, had low additive effects on PGC, ranging from –6.7 to –12.3% (Yang et al., 2021a,b). In this study, the two QTLs, *qPGC5* and *qPGC6*, had higher additive effects on PGC, ranging from –12.3 to –21.6% (Figure 5). The additive effects of *qPGC5* and *qPGC6* were almost twice that of the other four QTLs. Therefore, compared with *qPGC8.1*, *qPGC8.2*, *qPGC9* and *qPGC11*, *qPGC5* and *qPGC6* were major QTLs for rice chalkiness.

### *qPGC5* and *qPGC6* Had Additive Effects Enhanced by High Temperature

High temperature is an important factor for the formation of chalkiness. The emergence of high temperature stress during the grain-filling period leads to irregular grain-filling and obstacles to storage biosynthesis, resulting in the increase of rice chalkiness (Sreenivasulu et al., 2015; Edwards et al., 2017; Nevame et al., 2018). In the Guangzhou area of China, the air temperature in FCS is usually higher than that in SCS at the grain-filling stage (Supplementary Table 5; Yang et al., 2021a,b). Recently, two chalkiness QTLs *qPGC9* and *qPGC11* were detected and their additive effects on chalkiness in SCS were nearly double of that in FCS. Due to high temperature sensitivity, the effects of *qPGC9* and *qPGC11* were inhibited in FCS, resulting in higher chalkiness in FCS than that in SCS (Yang et al., 2021a). Unlike *qPGC9* and *qPGC11*, the other two QTLs for rice chalkiness, *qPGC8.1* and *qPGC8.2*, were not sensitive to high temperature and their additive effects on chalkiness were not significantly different between FCS and SCS (Yang et al., 2021b). In this study, the additive effects of *qPGC5* and *qPGC6* did not decrease but increased with high temperature, which made the chalkiness decreasing in FCS more than that in SCS (Figures 4, 5). Therefore, the response of chalkiness QTLs to high temperature is diverse. Under high temperature, the decreasing chalkiness ability of many QTLs was inhibited. This result revealed the reason why most cultivars usually exhibit higher chalkiness at high temperature. Fortunately, *qPGC5* and *qPGC6* were the major QTLs with additive effects enhanced by high temperature, which are rare QTLs for reducing rice chalkiness under heat stress. Therefore, *qPGC5* and *qPGC6* will be helpful to develop the rice cultivars with low chalkiness even under high temperature.

## Conclusion

Using the SSSLs 22–05 and 15–06, two QTLs for rice chalkiness, *qPGC5* and *qPGC6*, were fine-mapped by substitution mapping. *qPGC5* was located in the 876.5 kb interval of chromosome 5 and *qPGC6* was located in the 269.1 kb interval of chromosome 6. The additive effects of *qPGC5* and *qPGC6* on chalkiness were significantly greater in FCS than in SCS. The two QTLs were the major QTLs for chalkiness with additive effects enhanced by high temperature and their SSSL alleles were more effective in reducing chalkiness under high temperature condition in FCS. The discovery of these two QTLs provides new genetic resources for the development of new varieties with low chalkiness even under high temperature conditions.

## Data Availability Statement

The original contributions presented in this study are included in the article/Supplementary Material, further inquiries can be directed to the corresponding author/s.

## Author Contributions

GZ and SW conceptualized, designed, and supervised the work. WY conducted most of the experiments, analyzed the experimental data, and wrote the original draft. QH, JL, QT, XL, SL, HZ, SB, ZL, and GL performed a part of experiments. GZ analyzed the data and wrote the manuscript. All authors reviewed and approved the final manuscript.

## Conflict of Interest

The authors declare that the research was conducted in the absence of any commercial or financial relationships that could be construed as a potential conflict of interest.

## Publisher’s Note

All claims expressed in this article are solely those of the authors and do not necessarily represent those of their affiliated organizations, or those of the publisher, the editors and the reviewers. Any product that may be evaluated in this article, or claim that may be made by its manufacturer, is not guaranteed or endorsed by the publisher.

## Footnotes

1
www.wseen.com

2
https://archive.gramene.org/markers/

3
https://www.originlab.com

## References

[B1] Chen H. Zhao Z. Jiang L. Wan X. Liu L. Wu X. (2011). Molecular genetic analysis on percentage of grains with chalkiness in rice (*Oryza sativa* L.). *Afr. J. Biotechnol.* 10 6891–6903. 10.5897/AJB11.208

[B2] Chen L. Gao W. Chen S. Wang L. Zou J. Liu Y. (2016). High-resolution QTL mapping for grain appearance traits and co-localization of chalkiness-associated differentially expressed candidate genes in rice. *Rice* 9:48. 10.1186/s12284-016-0121-6 27659284 PMC5033801

[B3] Cheng F. Liu Y. Liu Z. Zhao N. Wang F. Zhang Q. (2007). Positional variations in chalky occurrence within a rice panicle and its relation to grain nutritional quality. *Aust. J. Agr. Res.* 58:95. 10.1071/AR06138

[B4] Duncan D. B. (1955). Multiple range and multiple F tests. *Biometrics* 11 1–42. 10.2307/3001478

[B5] Edwards J. D. Jackson A. K. McClung A. M. (2017). Genetic architecture of grain chalk in rice and interactions with a low phytic acid locus. *Field Crop. Res.* 205 116–123. 10.1016/j.fcr.2017.01.015

[B6] Eshed Y. Zamir D. (1995). An introgression line population of *Lycopersicon pennellii* in the cultivated tomato enables the identification and fine mapping of yield-associated QTL. *Genetics* 141 1147–1162. 10.1101/gad.9.21.2712 8582620 PMC1206837

[B7] Fitzgerald M. A. McCouch S. R. Hall R. D. (2009). Not just a grain of rice: the quest for quality. *Trends Plant Sci.* 14 133–139. 10.1016/j.tplants.2008.12.004 19230745

[B8] Fujita N. Yoshida M. Kondo T. Saito K. Utsumi Y. Tokunaga T. (2007). Characterization of SSIIIa-deficient mutants of rice: the function of SSIIIa and pleiotropic effects by SSIIIa deficiency in the rice endosperm. *Plant Physiol.* 144 2009–2023. 10.1104/pp.107.102533 17586688 PMC1949899

[B9] Gao Y. Liu C. Li Y. Zhang A. Dong G. Xie L. (2016). QTL analysis for chalkiness of rice and fine mapping of a candidate gene for *qACE9*. *Rice* 9:41. 10.1186/s12284-016-0114-5 27549111 PMC4993740

[B10] Gong J. Miao J. Zhao Y. Zhao Q. Feng Q. Zhan Q. (2017). Dissecting the genetic basis of grain shape and chalkiness traits in hybrid rice using multiple collaborative populations. *Mol. Plant* 10 1353–1356. 10.1016/j.molp.2017.07.014 28803900

[B11] Jiang L. Zhong H. Jiang X. Zhang J. Huang R. Liao F. (2021). Identification and pleiotropic effect analysis of *GSE5* on rice chalkiness and grain shape. *Front. Plant Sci.* 12:814928. 10.3389/fpls.2021.814928 35126437 PMC8810533

[B12] Kang H. Park S. Matsuoka M. An G. (2005). White-core endosperm *floury endosperm-4* in rice is generated by knockout mutations in the C4-type pyruvate orthophosphate dikinase gene (*OsPPDKB*). *Plant J.* 42 901–911. 10.1111/j.1365-313X.2005.02423.x 15941402

[B13] Kobayashi A. Sonoda J. Sugimoto K. Kondo M. Iwasawa N. Hayashi T. (2013). Detection and verification of QTLs associated with heat-induced quality decline of rice (*Oryza sativa* L.) using recombinant inbred lines and near-isogenic lines. *Breed. Sci.* 63 339–346. 10.1270/jsbbs.63.339 24273430 PMC3770562

[B14] Laborte A. G. Paguirigan N. C. Moya P. F. Nelson A. Sparks A. H. Gregorio G. B. (2015). Farmers’ preference for rice traits: insights from farm surveys in Central Luzon, Philippines, 1966-2012. *PLoS One* 10:e136562. 10.1371/journal.pone.0136562 26317505 PMC4552743

[B15] Li Y. Fan C. Xing Y. Jiang Y. Luo L. Sun L. (2011). Natural variation in *GS5* plays an important role in regulating grain size and yield in rice. *Nat. Genet.* 43 1266–1269. 10.1038/ng.977 22019783

[B16] Li Y. Fan C. Xing Y. Yun P. Luo L. Yan B. (2014). *Chalk5* encodes a vacuolar H^+^-translocating pyrophosphatase influencing grain chalkiness in rice. *Nat. Genet.* 46 398–404. 10.1038/ng.2923 24633159

[B17] Liu J. Chen J. Zheng X. Wu F. Lin Q. Heng Y. (2017). *GW5* acts in the brassinosteroid signalling pathway to regulate grain width and weight in rice. *Nat. Plants* 3:17043. 10.1038/nplants.2017.43 28394310

[B18] Liu X. Wang Y. Wang S. W. (2012). QTL analysis of percentage of grains with chalkiness in *Japonica* rice (*Oryza sativa*). *Genet. Mol. Res.* 11 717–724. 10.4238/2012.March.22.1 22576829

[B19] Lou G. Chen P. Zhou H. Li P. Xiong J. Wan S. (2021). *FLOURY ENDOSPERM19* encoding a class I glutamine amidotransferase affects grain quality in rice. *Mol. Breed.* 41:36. 10.1007/s11032-021-01226-z PMC10236042 37309330

[B20] McCouch S. R. Cho Y. G. Yano M. Paul E. Blinstrub M. Mor-ishima H. (1997). II. Report from coordinators. (1) Report on QTL nomenclature. *Rice Genet. Newsl.* 14 11–12.

[B21] McCouch S. R. Teytelman L. Xu Y. Lobos K. B. Clare K. Walton M. (2002). Development and mapping of 2240 new SSR markers for rice (*Oryza sativa* L.). *DNA Res.* 9 199–207. 10.1093/dnares/9.6.257 12597276

[B22] Misra G. Anacleto R. Badoni S. Butardo V. Molina L. Graner A. (2019). Dissecting the genome-wide genetic variants of milling and appearance quality traits in rice. *J. Exp. Bot.* 70 5115–5130. 10.1093/jxb/erz256 31145789 PMC6793453

[B23] Misra G. Badoni S. Parween S. Singh R. K. Leung H. Ladejobi O. (2020). Genome-wide association coupled gene to gene interaction studies unveil novel epistatic targets among major effect loci impacting rice grain chalkiness. *Plant Biotechnol. J.* 19 910–925. 10.1111/pbi.13516 33220119 PMC8131057

[B24] Nevame A. Y. M. Emon R. M. Malek M. A. Hasan M. M. Alam M. A. Muharam F. M. (2018). Relationship between high temperature and formation of chalkiness and their effects on quality of rice. *Biomed Res. Int.* 2018 1–18. 10.1155/2018/1653721 30065932 PMC6051336

[B25] Peng B. Wang L. Fan C. Jiang G. Luo L. Li Y. (2014). Comparative mapping of chalkiness components in rice using five populations across two environments. *BMC Genet.* 15:49. 10.1186/1471-2156-15-49 24766995 PMC4021085

[B26] Quero G. Gutiérrez L. Monteverde E. Blanco P. Pérez De Vida F. Rosas J. (2018). Genome-wide association study using historical breeding populations discovers genomic regions involved in high-quality rice. *Plant Genome* 11:170076. 10.3835/plantgenome2017.08.0076 30512035 PMC12810139

[B27] She K. Kusano H. Koizumi K. Yamakawa H. Hakata M. Imamura T. (2010). A novel factor *FLOURY ENDOSPERM2* is involved in regulation of rice grain size and starch quality. *Plant Cell* 22 3280–3294. 10.1105/tpc.109.070821 20889913 PMC2990130

[B28] Song X. Huang W. Shi M. Zhu M. Lin H. (2007). A QTL for rice grain width and weight encodes a previously unknown RING-type E3 ubiquitin ligase. *Nat. Genet.* 39 623–630. 10.1038/ng2014 17417637

[B29] Sreenivasulu N. Butardo V. M. Misra G. Cuevas R. P. Anacleto R. Kavi Kishor P. B. (2015). Designing climate-resilient rice with ideal grain quality suited for high-temperature stress. *J. Exp. Bot.* 66 1737–1748. 10.1093/jxb/eru544 25662847 PMC4669556

[B30] Tan Q. Zou T. Zheng M. Ni Y. Luan X. Li X. (2020). Substitution mapping of the major quantitative trait loci controlling stigma exsertion rate from *Oryza glumaepatula*. *Rice* 13:37. 10.1186/s12284-020-00397-1 32519122 PMC7283377

[B31] Tan Y. F. Xing Y. Z. Li J. X. Yu S. B. Xu C. G. Zhang Q. (2000). Genetic bases of appearance quality of rice grains in Shanyou 63, an elite rice hybrid. *Theor. Appl. Genet.* 101 823–829. 10.1007/s001220051549 22665200

[B32] Temnykh S. Park W. D. Ayres N. Cartinhour S. Hauck N. Lipovich L. (2000). Mapping and genome organization of microsatellite sequences in rice (*Oryza sativa* L.). *Theor. Appl. Genet.* 100 697–712. 10.1007/s001220051342

[B33] Wada T. Miyahara K. Sonoda J. Tsukaguchi T. Miyazaki M. Tsubone M. (2015). Detection of QTLs for white-back and basal-white grains caused by high temperature during ripening period in japonica rice. *Breed. Sci.* 65 216–225. 10.1270/jsbbs.65.216 26175618 PMC4482171

[B34] Wan X. Y. Wan J. M. Weng J. F. Jiang L. Bi J. C. Wang C. M. (2005). Stability of QTLs for rice grain dimension and endosperm chalkiness characteristics across eight environments. *Theor. Appl. Genet.* 110 1334–1346. 10.1007/s00122-005-1976-x 15809851

[B35] Wang E. Wang J. Zhu X. Hao W. Wang L. Li Q. (2008). Control of rice grain-filling and yield by a gene with a potential signature of domestication. *Nat. Genet.* 40 1370–1374. 10.1038/ng.220 18820698

[B36] Wang S. Li S. Liu Q. Wu K. Zhang J. Wang S. (2015). The *OsSPL16-GW7* regulatory module determines grain shape and simultaneously improves rice yield and grain quality. *Nat. Genet.* 47 949–954. 10.1038/ng.3352 26147620

[B37] Wang S. Wu K. Yuan Q. Liu X. Liu Z. Lin X. (2012). Control of grain size, shape and quality by *OsSPL16* in rice. *Nat. Genet.* 44 950–954. 10.1038/ng.2327 22729225

[B38] Wang X. Pang Y. Wang C. Chen K. Zhu Y. Shen C. (2017). New candidate genes affecting rice grain appearance and milling quality detected by genome-wide and gene-based association analyses. *Front. Plant Sci.* 7:1998. 10.3389/fpls.2016.01998 28101096 PMC5209347

[B39] Wang Z. Y. Zheng F. Q. Shen G. Z. Gao J. P. Snustad D. P. Li M. G. (1995). The amylose content in rice endosperm is related to the post-transcriptional regulation of the *waxy* gene. *Plant J.* 7 613–622. 10.1046/j.1365-313X.1995.7040613.x 7742858

[B40] Woo M. Ham T. Ji H. Choi M. Jiang W. Chu S. (2008). Inactivation of the *UGPase1* gene causes genic male sterility and endosperm chalkiness in rice (*Oryza sativa* L.). *Plant J.* 54 190–204. 10.1111/j.1365-313X.2008.03405.x 18182026 PMC2327258

[B41] Wu B. Xia D. Zhou H. Cheng S. Wang Y. Li M. (2021). Fine mapping of *qWCR7*, a grain chalkiness QTL in rice. *Mol. Breed.* 41:68. 10.1007/s11032-021-01260-x PMC10236040 37309362

[B42] Wu B. Yun P. Zhou H. Xia D. Gu Y. Li P. (2022). Natural variation in *WHITE-CORE RATE 1* regulates redox homeostasis in rice endosperm to affect grain quality. *Plant Cell* 34 1912–1932. 10.1093/plcell/koac057 35171272 PMC9048946

[B43] Wu M. Ren Y. Cai M. Wang Y. Zhu S. Zhu J. (2019). Rice *FLOURY ENDOSPERM10* encodes a pentatricopeptide repeat protein that is essential for the trans-splicing of mitochondrial nad1 intron 1 and endosperm development. *New Phytol.* 223 736–750. 10.1111/nph.15814 30916395

[B44] Yamakawa H. Ebitani T. Terao T. (2008). Comparison between locations of QTLs for grain chalkiness and genes responsive to high temperature during grain filling on the rice chromosome map. *Breed. Sci.* 58 337–343. 10.1270/jsbbs.58.337 26081539

[B45] Yang W. Guo Z. Huang C. Duan L. Chen G. Jiang N. (2014). Combining high-throughput phenotyping and genome-wide association studies to reveal natural genetic variation in rice. *Nat. Commun.* 5:5087. 10.1038/ncomms6087 25295980 PMC4214417

[B46] Yang W. Liang J. Hao Q. Luan X. Tan Q. Lin S. (2021a). Fine mapping of two grain chalkiness QTLs sensitive to high temperature in rice. *Rice* 14:33. 10.1186/s12284-021-00476-x 33792792 PMC8017073

[B47] Yang W. Xiong L. Liang J. Hao Q. Luan X. Tan Q. (2021b). Substitution mapping of two closely linked QTLs on chromosome 8 controlling grain chalkiness in rice. *Rice* 14:85. 10.1186/s12284-021-00526-4 34601659 PMC8487414

[B48] Yang W. Xu P. Zhang J. Zhang S. Li Z. Yang K. (2022). *OsbZIP60*-mediated unfolded protein response regulates grain chalkiness in rice. *J. Genet. Genomics* 49 414–426. 10.1016/j.jgg.2022.02.002 35189403

[B49] Yun P. Zhu Y. Wu B. Gao G. Sun P. Zhang Q. (2016). Genetic mapping and confirmation of quantitative trait loci for grain chalkiness in rice. *Mol. Breed.* 36:162. 10.1007/s11032-016-0600-x

[B50] Zhang G. (2019). The platform of breeding by design based on the SSSL library in rice. *Hereditas* 41 754–760. 10.16288/j.yczz.19-105 31447426

[B51] Zhang G. (2021). Target chromosome-segment substitution: a way to breeding by design in rice. *Crop J.* 9 658–668. 10.1016/j.cj.2021.03.001

[B52] Zhang L. Ren Y. Lu B. Yang C. Feng Z. Liu Z. (2016). *FLOURY ENDOSPERM7* encodes a regulator of starch synthesis and amyloplast development essential for peripheral endosperm development in rice. *J. Exp. Bot.* 67 633–647. 10.1093/jxb/erv469 26608643 PMC4737065

[B53] Zhao D. Li Q. Zhang C. Zhang C. Yang Q. Pan L. (2018). *GS9* acts as a transcriptional activator to regulate rice grain shape and appearance quality. *Nat. Commun.* 9:1240. 10.1038/s41467-018-03616-y 29588443 PMC5869696

[B54] Zhao X. Daygon V. D. McNally K. L. Hamilton R. S. Xie F. Reinke R. F. (2016). Identification of stable QTLs causing chalk in rice grains in nine environments. *Theor. Appl. Genet.* 129 141–153. 10.1007/s00122-015-2616-8 26498441

[B55] Zhao X. Zhou L. Ponce K. Ye G. (2015). The usefulness of known genes/QTLs for grain quality traits in an indica population of diverse breeding lines tested using association analysis. *Rice* 8:29. 10.1186/s12284-015-0064-3 26391157 PMC4577492

[B56] Zhou H. Yang W. Ma S. Luan X. Zhu H. Wang A. (2020). Unconditional and conditional analysis of epistasis between tillering QTLs based on single segment substitution lines in rice. *Sci. Rep.* 10:15912. 10.1038/s41598-020-73047-7 32985566 PMC7523009

[B57] Zhou L. Chen L. Jiang L. Zhang W. Liu L. Liu X. (2009). Fine mapping of the grain chalkiness QTL *qPGWC-7* in rice (*Oryza sativa* L.). *Theor. Appl. Genet.* 118 581–590. 10.1007/s00122-008-0922-0 19020855

