# Supplementary material for: Fine Mapping of Two Major Quantitative Trait Loci for Rice Chalkiness With High Temperature-Enhanced Additive Effects
Source: Front Plant Sci. 2022 Jun 30;13:957863. doi: 10.3389/fpls.2022.957863 (PMC9280674; doi:10.3389/fpls.2022.957863)
Supplement: Supplementary file 2 [file Image_1.pdf]

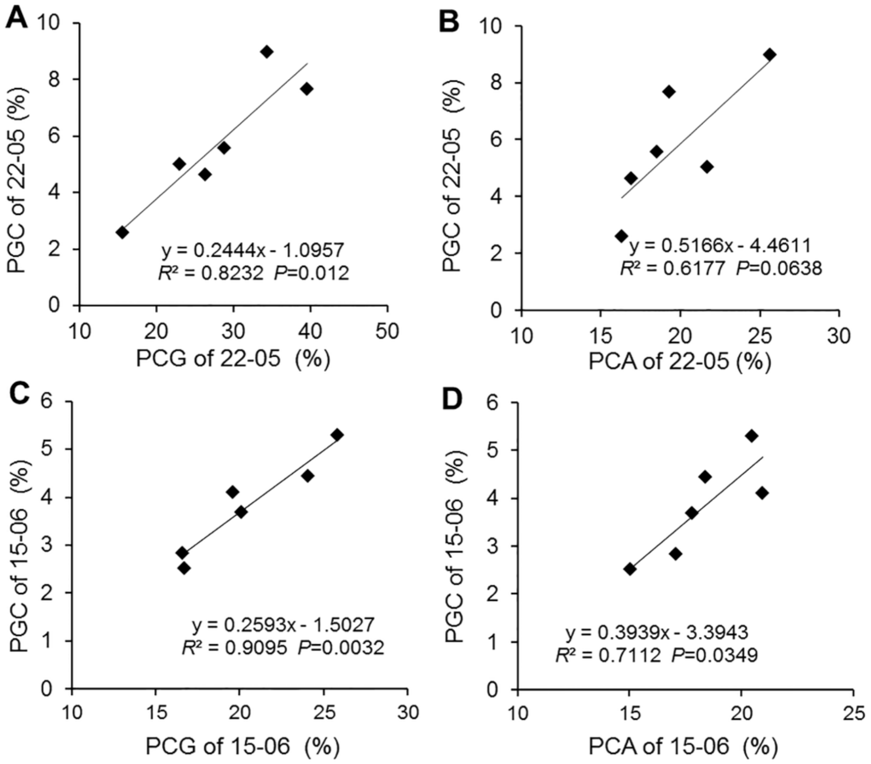

Supplementary Figure 1 Regression correlation between PCG and PGC and between PCA and PGC in SSSLs 22-05 and 15-06. R^2, represents the percentage of x contribution to y phenotype variation. PCG, percentage of chalky grain. PCA, percentage of chalky area. PGC, percentage of grain chalkiness.
